# Supplementary material for: Reshaping organellar translation and tRNA metabolism: the consequences of photosynthesis loss and massive horizontal gene transfer
Source: Mol Biol Evol. 2026 Mar 23;43(4):msag077. doi: 10.1093/molbev/msag077 (PMC13128298; doi:10.1093/molbev/msag077)
Supplement: msag077_Supplementary_Data [file msag077_supplementary_data.zip › Supplementary Note 1.docx]

**Supplementary Note 1**

**CTAB-based RNA Extraction Protocol**

1. **Buffer Preparation:** 100 mL of extraction buffer was prepared according to the specific formulation:
   - 300 mM Tris-HCl (pH 8.0)
   - 2% (w/v) CTAB
   - 2% (w/v) PVP-40
   - 2 M NaCl
   - 25 mM EDTA
   - 2% (v/v) β-mercaptoethanol

All stock solutions were prepared using diethyl pyrocarbonate (DEPC)-treated water and autoclaved

1. The buffer was prepared in a glass bottle containing a magnetic stir bar (previously autoclaved). Liquid components were added first, followed by solid components. The volume was adjusted with DEPC-treated water (excluding β-mercaptoethanol). The pH of the CTAB solution was adjusted to 7.0–7.5 using HCl (<1 mL per 50 mL of buffer) in a separate autoclaved container.
2. The extraction buffer was dissolved with heating and stirring (30 min, 150°C setting, 500 rpm) and subsequently maintained at 30°C.
3. Immediately prior to use, 200 μL of β-mercaptoethanol was added to every 10 mL of extraction buffer.
4. 10 mL of the complete extraction buffer was added to 50 mL tubes containing the sample. The mixture was homogenized by inversion and vortexing.
5. Samples were incubated under two conditions: one aliquot at 30°C and another at 65°C for 20 min. Tubes were agitated vigorously every 3 min during incubation.
6. 8 mL of cold Chloroform:Isoamyl alcohol (24:1) was added. The tubes were mixed gently until two phases were observed. Samples were centrifuged at 6°C for 20 min at 7,000 rpm.
   - *Note: From this step onward, all procedures were performed on ice.*
7. The supernatant (approximately 8 mL) was transferred to a new cold 15 mL Falcon tube.
8. Extraction was repeated by adding 7 mL of Chloroform:Isoamyl alcohol (24:1). Samples were centrifuged at 4°C for 20 min at 7,000 rpm.
9. The supernatant (7 mL) was transferred to a new cold tube. Precipitation was induced by adding 0.1 volumes (700 μL) of cold 3 M NaOAc (pH 5.2) and 0.6 volumes (4.2 mL) of cold isopropanol. The solution was mixed carefully.
10. Samples were incubated at -80°C for 15 min.
11. Samples were centrifuged at 4°C for 30 min at 7,000 rpm to pellet the RNA.
12. The supernatant was carefully decanted into a beaker. The tube was briefly held vertically, and residual supernatant was removed using a micropipette without disturbing the pellet. The pellet was air-dried for 5–10 min on ice.
13. The pellet was washed with 1 mL of cold 70% ethanol (diluted with DEPC-treated water). Samples were centrifuged at 4°C for 5 min at 13,200 rpm. The supernatant was discarded, and residual liquid was removed by pipetting. The pellet was air-dried for approximately 10 min on ice.
14. The RNA pellet was resuspended in 50 μL of DEPC-treated water by gentle mixing. Resuspension was allowed to proceed on ice for 2 hours.
15. RNA samples were stored at -80°C.
